# Global Research Trends on Polyhydroxyalkanoate-Producing Microorganisms: A Bibliometric and Scientometric Analysis of Sustainable Bioplastic Biotechnology

**DOI:** 10.3390/microorganisms14071550

**Published:** 2026-07-15

**Authors:** Magaly De La Cruz-Noriega, Ana María Sabogal Vargas, Walter Rojas Villacorta, Waldo Salvatierra Espinola, Claudio Quiñones-Cerna

**Affiliations:** 1 Grupo de Investigación en Biotecnología y Bioingeniería, Institutos y Centro de Investigación, Universidad Cesar Vallejo, Trujillo 13001, Peru; 2 Escuela de Medicina, Facultad de Ciencias de la Salud, Universidad Cesar Vallejo, Trujillo 13001, Peru; asabogal@ucv.edu.pe; 3 Grupo de Biotecnología Microbiana y Vegetal, Universidad César Vallejo, Trujillo 13001, Peru; wrojasv@ucv.edu.pe; 4 Escuela de Enfermería, Facultad de Ciencias de la Salud, Universidad Cesar Vallejo, Trujillo 13001, Peru; wsavatierraes@ucvvirtual.edu.pe; 5 Laboratorio de Biotecnología e Ingeniería Genética, Universidad Nacional de Trujillo, Trujillo 13001, Peru; cquinonesc@unitru.edu.pe

**Keywords:** polyhydroxyalkanoates (PHAs), bioplastics, sustainable biotechnology, metabolic engineering

## Abstract

This scientometric and bibliometric study analyzes global research trends on polyhydroxyalkanoate (PHA)-producing microorganisms and their applications in bio-based packaging between 2014 and 2025. Using the Scopus database and advanced tools such as Bibliometrix in RStudio and VOSviewer, scientific output and international collaboration networks were evaluated. The results demonstrate exponential growth in publications, driven by the urgent need to mitigate the petrochemical plastics crisis and develop biodegradable alternatives within the circular economy. Multidisciplinary analysis reveals a thematic shift from basic physiological and taxonomic studies towards complex applications in metabolic engineering, synthetic biology, the optimization of low-cost substrates such as industrial effluents, multi-omics tools, gene editing with CRISPR-Cas9, and, as an emerging exploratory approach, quantum modeling to optimize cell performance. Despite significant progress, critical technological gaps were identified related to challenges in downstream processing, the management of mixed microbial communities, and insufficient funding for the characterization of physicochemical and biocompatibility properties. It is concluded that, to ensure the commercial scalability and sustainability of PHAs, future research must prioritize overcoming these economic and technological bottlenecks, fostering strategic collaboration between academia and the biotechnology industry.

## 1. Introduction

The global crisis caused by petrochemical plastics has reached alarming levels: in 2019 alone, global production reached 368 million metric tons, and estimates indicate that it already surpassed the 600 million barrier by 2025 [1]. The real problem begins with its final destination, since up to the year 2015, 6300 million metric tons of plastic waste had accumulated, of which a meager 9% managed to be recycled; in contrast, 79% ended up buried in landfills or abandoned in nature, a concerning reality that causes about 10 million tons of synthetic plastics to leak into the oceans every year [2]. Given this scenario, polyhydroxyalkanoates (PHAs) emerge as a biodegradable alternative with enormous potential, backed by a market that was projected to reach 100 million dollars by 2024 [3]. However, the major bottleneck for their definitive leap to industry remains the cost, as producing a single kilogram of PHA costs between 2 and 6 dollars, a very high figure compared to the competitive 1 or 2 dollars of traditional plastics [4]. The analysis of scientific production reflects this interest, with exponential growth since 1999 until reaching a total of 1227 publications by 2024, evolving from basic physiological studies toward phases of metabolic engineering and applied biotechnology [5]. The importance of researching polyhydroxyalkanoate (PHA)-producing microorganisms lies in their enormous potential to mitigate the environmental crisis caused by synthetic plastics, an alarming problem considering that only 9% of this waste is recycled and that its mass production could consume 20% of the world’s petroleum by the year 2050 [2]. In this context, PHAs position themselves as a fundamental alternative thanks to being 100% biodegradable and biocompatible [5,6].

**Figure 1:**
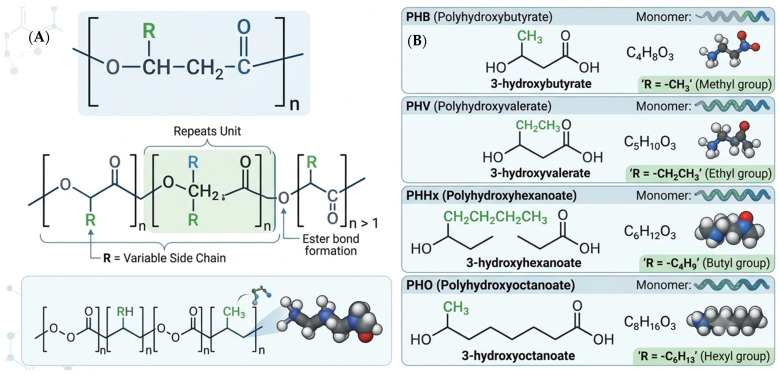
General chemical structure of polyhydroxyalkanoates (PHAs). (**A**) Basic repeating unit: [-O-CH(R)-CH_2_-C(=O)-]_n_. (**B**) Examples of PHA monomers with different side chains (R): PHB (R = CH_3_), PHV (R = CH_2_CH_3_), PHHx (R = C_4_H_9_), and PHO (R = C_6_H_13_).

Chemically, polyhydroxyalkanoates are linear polyesters composed of (R)-3-hydroxyalkanoic acid monomers linked by ester bonds between the carboxyl group of a monomer and the hydroxyl group of the adjacent unit [7] (Figure 1). The general structure corresponds to [-O-CH(R)-CH_2_-C(=O)-]n, where the R side chain can vary from a methyl group (in poly(3-hydroxybutyrate), PHB) to longer alkyl substituents up to C_13_, depending on the substrate and the microbial strain used [8,9]. This structural versatility is key to understanding differences in physicochemical properties, as the length and chemical nature of the side chain directly influence the crystallinity, glass transition temperature (Tg), and melting point (Tm) of the polymer [10,11]. Short-chain PHAs (scl-PHAs), such as PHB with R = CH_3_, exhibit high crystallinity (60–80%) and rigidity, making them suitable for rigid packaging and thermoformed containers [12]. In contrast, medium-chain PHAS (MCL-PHAs), such as those produced by *Pseudomonas putida* with R = C_3_H_7_ to C_9_H_19_, exhibit lower crystallinity (20–40%) and greater flexibility, ideal for films, elastomers, and coatings [13]. The presence of unsaturated or branched side chains, as reported in the monomeric composition of *Klebsiella pneumoniae* P6 [14], allows for chemical modifications following polymerization, such as grafting or crosslinking, significantly expanding the spectrum of applications of these biopolymers, as shown.

**Figure 2:**
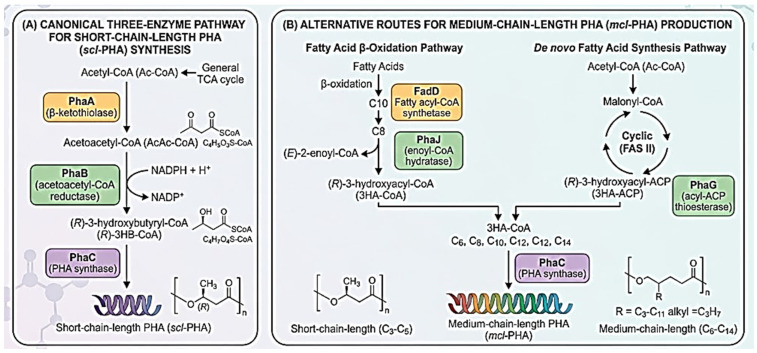
Simplified scheme of the polyhydroxyalkanoate (PHA) biosynthetic pathway. (**A**) Canonical three-enzyme pathway for short-chain-length PHA (scl-PHA) synthesis from acetyl-CoA: PhaA (β-ketothiolase), PhaB (acetoacetyl-CoA reductase), and PhaC (PHA synthase). (**B**) Alternative routes for medium-chain-length PHA (mcl-PHA) production via fatty acid β-oxidation and de novo fatty acid synthesis, involving key enzymes such as PhaG, PhaJ, and FadD. Abbreviations: Ac-CoA, acetyl-CoA; AcAc-CoA, acetoacetyl-CoA; (R)-3HB-CoA, (R)-3-hydroxybutyryl-CoA; 3HA-CoA, (R)-3-hydroxyacyl-CoA; TCA, tricarboxylic acid cycle.

The biosynthesis of polyhydroxyalkanoates in bacteria follows a conserved metabolic pathway that begins with central carbon metabolism. The canonical pathway for short-chain PHAs (scl-PHAs), such as poly(3-hydroxybutyrate) (PHB), comprises three enzymatic steps (Figure 2) [15]. First, two acetyl-CoA molecules are condensed by β-ketothiolase (PhaA) to form acetoacetyl-CoA. Second, acetoacetyl-CoA reductase (PhaB) reduces this intermediate to (R)-3-hydroxybutyryl-CoA using NADPH as a cofactor. Finally, PHA synthase (PhaC) polymerizes (R)-3-hydroxybutyryl-CoA monomers into high-molecular-weight PHB granules [3,7]. For medium-chain-length PHAs (mcl-PHAs), such as those produced by *Pseudomonas putida*, the biosynthetic pathway may involve alternative pathways, including the fatty acid β-oxidation cycle and the de novo fatty acid synthesis pathway [16,17,18]. In these pathways, (R)-3-hydroxyacyl-CoA samples with longer side chains (C_6_–C_14_) are generated and subsequently polymerized by class II PHA synthases (PhaC1 and PhaC2) [19,20]. Key accessory enzymes include PhaG ((R)-3-hydroxyacyl-ACP:CoA transacylase), which channels the intermediates of fatty acid synthesis, and PhaJ (((R)-enoyl-CoA specific hydratase), which participates in the β-oxidation pathway [17,21]. Understanding this pathway is critical to understanding the metabolic engineering strategies discussed in this scientometric review. Strategic manipulation of these enzymes, through gene overexpression, deletion, or CRISPR-Cas9-mediated editing, allows for redirecting carbon flux towards PHA synthesis, the incorporation of new monomers, and improved overall yields [22,23,24]. As shown in Figure 2, the supply of precursors of the TCA cycle (via succinyl-CoA) and the glycolytic pathway can be redirected to increase the availability of acetyl-CoA, the main building block for PHA biosynthesis [25,26].

The biochemical foundations of PHA biosynthesis were established through the pioneering work of researchers such as Steinbüchel and Rehm, who elucidated that the molecular mechanisms of PHA synthases are the key enzymes responsible for polymer formation and the genetic organization of PHA biosynthetic operons [27]. Subsequent studies by Stubbe and colleagues provided detailed insights into the catalytic mechanism of PHA synthases, including the processes of initiation, elongation, and termination, as well as the role of accessory proteins such as phasins in granule formation and homeostasis [28,29,30]. These foundational contributions established the framework for metabolic engineering approaches that enable the rational design of microbial strains for enhanced PHA production.

However, their definitive leap to an industrial scale still clashes with a major economic barrier: their production costs can be up to double those of conventional synthetic polymers [4,14]. To overcome this obstacle, current strategies focus on utilizing low-cost substrates, such as agricultural waste and industrial effluents [14,31], combined with the optimization of biotechnological processes through advanced statistical tools like response surface methodology [32]. In parallel, metabolic engineering and the use of cutting-edge technologies like CRISPR-Cas9 are allowing the custom design of microbial strains to achieve much more competitive yields [33]. In this dynamic scenario, scientometric analysis becomes a vital tool, as it allows mapping global trends and accurately identifying those technological gaps that still hinder the scalability of this sustainable biotechnology [5].

Scientific research on polyhydroxyalkanoate (PHA)-producing microorganisms has experienced a highly robust evolution, consolidating itself today thanks to the analysis of global trends and the latest advances in data mining. In this regard, Al Zamzami et al. (2025) executed an exhaustive scientometric analysis covering seven decades of research (1955–2024), revealing how scientific production skyrocketed exponentially to reach 1227 publications; in fact, their most outstanding finding was identifying a clear transition from basic bacterial physiology toward complex industrial applications in bioremediation and the synthesis of high-value-added compounds [5]. Complementarily, Vuong et al. (2021) provided a valuable technical perspective through the massive genomic mining of databases, successfully categorizing the diversity of PHA synthase enzyme genotypes across the Bacteria and Archaea domains; this study allowed a thorough mapping of the trends inherent to producing microorganisms, facilitating the identification of under-explored taxa with high biotechnological potential for producing unique monomers [2]. For their part, Sabri et al. (2024) reviewed fermentation strategies for the bioconversion of toxic waste into bioplastics, highlighting that the integration of mixed microbial communities and the design of specific feeding strategies are currently the most effective methods to maximize PHA accumulation and efficiently manage industrial waste [9]. All these antecedents demonstrate how vital it is to use scientometric tools, not only to organize the vast volume of available scientific information but also to precisely detect those technological gaps that still hinder the commercial scalability of sustainable bioplastics.

In the field of bioprospecting for efficient microorganisms, Ansari et al. [14] in 2025, carried out a study focused on the isolation and identification of bacterial strains from palm oil mill effluents (POMEs), an abundant industrial waste with a high content of organic compounds. The most outstanding finding of this research was the characterization of the strain *Klebsiella pneumoniae* P6, which exhibited a superior biosynthesis capacity for polyhydroxyalkanoates (PHAs) when using oleic acid as a substrate, reaching a fluorescence intensity of 16,698.4 A.U. in screening tests. Analysis using gas chromatography and mass spectrometry revealed an exceptionally diverse monomeric composition, which includes short-, medium-, and long-chain monomers (C4, C14, C16, and C18), highlighting the presence of unsaturated derivatives such as 11-octadecenoic acid methyl ester. This result is of great relevance for global research trends, since the incorporation of unsaturated monomers allows subsequent chemical modification of the polymer (such as grafting or cross-linking) and significantly improves its processability by reducing crystallinity and increasing flexibility compared to conventional PHAs, which tend to be brittle. This type of background reinforces the importance of scientometric analyses to map the scientific transition toward the use of low-cost raw materials and the identification of under-documented microbial taxa, determining factors for reducing production costs and facilitating the scalability of bioplastics biotechnology within a circular economy.

The importance of conducting a bibliometric review on polyhydroxyalkanoate (PHA)-producing microorganisms lies in its ability to systematically organize the extensive scientific literature on the subject, map the evolution of knowledge, and identify key technological gaps for the advancement of sustainable biotechnology. To this end, the Scopus database is essential due to its rigorous and broad coverage across interdisciplinary fields, as well as its robust tools for citation tracking and author disambiguation. By integrating advanced software tools, such as the Bibliometrix package (V 5.4.1) in Rstudio (2022.12.0+353) and VOSviewer (V 1.6.21), precise scientometric analyses can be performed that quantify scientific productivity and visualize complex global collaboration networks. These methodologies facilitate the early detection of emerging research fronts and dominant themes, allowing for the observation of critical transitions in the discipline, such as the shift from basic physiological studies to more complex phases of metabolic engineering and synthetic biology. This robust methodological approach supports strategic decision-making and fosters synergistic collaboration between academia and industry, key factors for accelerating the commercial scalability of these bioplastics and ensuring their effective integration into a circular economy. However, despite these advances, a gap remains in the scientometric mapping of global trends specifically focused on PHA producing microorganisms. Systematic analyses that delve into the technological gaps related to the use of understudied taxa and the management of mixed microbial communities to optimize their industrial scalability are lacking.

The objective of this scientometric review is to quantify the evolution and exponential growth of global scientific output on polyhydroxyalkanoate (PHA)producing microorganisms between 2014 and 2025, and to analyze its temporal distribution to identify the development phases of this biotechnological field. Furthermore, it seeks to identify and evaluate key players, including the most productive authors, the highest-impact journals, and the leading institutions driving the advancement of sustainable bioplastics. Another fundamental aspect is to map the transition of research topics by analyzing the co-occurrence of keywords to visualize the evolution from basic taxonomic and physiological studies to complex applications in metabolic engineering, synthetic biology, and the circular economy. Similarly, the review aims to analyze the structure of global collaboration, examining co-authorship networks among countries such as India, the United Kingdom, and China, in order to identify centers of influence and the level of international integration. This study will analyze global trends and technological gaps in research on PHA-producing microorganisms and their applications in biological packaging using scientometric analysis to assess their industrial scalability. Finally, the study aims to identify specific technological gaps and knowledge gaps, such as downstream processing challenges, insufficient funding for characterizing physicochemical properties, and limited exploration of certain taxa, in order to propose future research directions that ensure the industrial scalability and sustainability of PHAs.

## 2. Materials and Methods

### 2.1. Research Design and Data Source

This study was based on a comprehensive bibliometric and scientometric analysis conducted to map global research trends on polyhydroxyalkanoate (PHA)-producing microorganisms and their applications in sustainable bio-packaging. This methodological approach was selected for its proven effectiveness in systematizing large volumes of the scientific literature, identifying hidden collaboration networks, and detecting emerging research niches within rapidly expanding interdisciplinary fields [5]. The methodological choices made in this study including the use of Scopus as the sole data source, the restriction to Open Access publications, and the selection of the 2014–2025time window were guided by the need for data quality, reproducibility, and feasibility. Scopus was selected for its comprehensive coverage of the peer-reviewed literature, rigorous indexing, and robust citation tracking capabilities. Open Access filtering was applied to ensure unrestricted access to complete metadata and to facilitate reproducibility of the analysis. The 2014–2025 period was chosen to capture the most recent scientific output, coinciding with the exponential growth phase of PHA research.

### 2.2. Search Strategy and Equation Used

The retrieval of the documents was carried out using a structured query designed to capture the multidisciplinary nature of PHA research, while simultaneously ensuring a high degree of specificity toward microbial systems. The search equation applied in Scopus was the following: (TITLE-ABS-KEY (“polyhydroxyalkanoate” OR “PHA” OR “biopolymer” OR “bioplastic”) AND TITLE-ABS-KEY (“microorganism” OR “bacteria” OR “fungi” OR “yeast”) AND TITLE-ABS-KEY (“sustainable” OR “eco-friendly” OR “renewable” OR “green”) AND TITLE-ABS-KEY (“biotechnology” OR “bioprocess” OR “fermentation” OR “production”) AND TITLE-ABS-KEY (“applications” OR “uses” OR “properties” OR “characteristics”)) AND (EXCLUDE (EXACTKEYWORD, “Nonhuman”) OR EXCLUDE (EXACTKEYWORD, “Article”) OR EXCLUDE (EXACTKEYWORD, “Review”) OR EXCLUDE (EXACTKEYWORD, “Human”)). This query combined Boolean operators to integrate five major conceptual axes: biopolymer terminology, microbial agents, sustainability criteria, biotechnological processes, and end-use applications. Subsequently, restrictive filters were applied to limit the results exclusively to original articles and reviews published in the English language, under Open Access, within the defined timeframe. The decision to include only open-access publications guaranteed the reproducibility of the study and unrestricted access to the complete metadata.

**Figure 3:**
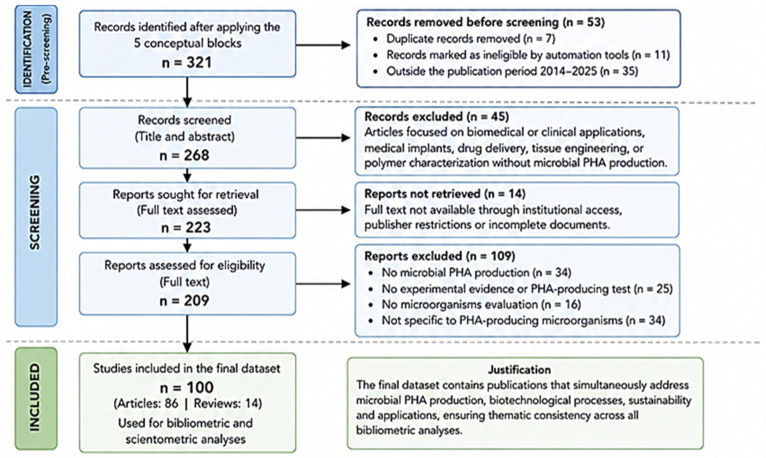
Flowchart of the search, refinement, and analysis process.

The sequential filtering process shown in Figure 3 was designed to progressively improve the thematic specificity of the bibliometric dataset. The initial search intentionally captured a broad multidisciplinary literature on polyhydroxyalkanoates. Subsequent screening removed duplicate records, documents outside the selected publication period, and publications lacking complete metadata. Title and abstract screening excluded studies focused exclusively on biomedical applications or unrelated polymer research. Full-text eligibility assessment further removed publications that did not investigate microbial PHA production, microbial producers, or properties relevant to sustainable bioplastic applications. Consequently, the final dataset consisted of studies directly aligned with the objectives of this scientometric analysis, thereby ensuring consistency, reproducibility and thematic relevance of the bibliometric results.

### 2.3. Data Management and Preprocessing

Following the execution of the search equation and the application of the five conceptual blocks, a total of 321 documents were retrieved from Scopus. After the removal of duplicates (*n* = 7), records marked as ineligible by automation tools (*n* = 11), and documents outside the 2014–2025 publication period (*n* = 35), 268 unique records remained for title and abstract screening. Following fulltext assessment, 209 reports were evaluated for eligibility, resulting in a final corpus of 100 documents: 86 original research articles (86%) and 14 review articles (14%). This final dataset was used for all bibliometric and scientometric analyses.

### 2.4. Analytical Tools and Impact Indicators

For the quantitative processing and visualization of scientific networks, a complementary set of specialized tools was used. VOSviewer (version 1.6.15) was utilized to construct keyword co-occurrence maps and co-authorship networks among countries, applying the association strength normalization method to properly weight the relationships between nodes [34]. Concurrently, the Bibliometrix package (V 5.4.1) within RStudio (2022.12.0+353) software was implemented to calculate advanced indicators, such as the h-index of authors and institutions, Lotka’s law for author productivity, and Bradford’s law for journal dispersion [35]. Production metrics (total number of documents and annual growth) and impact metrics (average citations per document and journal impact factor according to the Journal Citation Reports quartiles) were evaluated. This dual approach allowed not only for the quantification of the scientific leadership of countries like India and the United Kingdom but also for the evaluation of the quality and influence of the most cited research.

### 2.5. Network Visualization and Thematic Evolution

The final phase of the methodology consisted of generating graphical representations to facilitate the interpretation of the structural and dynamic patterns of the field. The network maps were constructed using VOSviewer with default parameters, where the thickness of the links indicates the strength of the co-occurrence relationships and the colors represent the algorithmically identified thematic clusters. Furthermore, temporal overlays (overlay maps) were applied to track the evolution of research topics throughout the study period, highlighting the shift from classic physiological approaches toward cutting-edge areas such as CRISPR-Cas9 gene editing and, as an emerging exploratory direction, quantum-like metabolic modeling.

### 2.6. Gap Score Calculation Methodology

To systematically identify and prioritize research gaps in the field of PHA-producing microorganisms, a quantitative Gap_Score indicator was developed and applied to the keyword co-occurrence and thematic evolution analyses. The Gap_Score was calculated using a multi-component formula that integrates three dimensions of research underrepresentation:

Gap_Score = (F × 0.4) + (D × 0.3) + (C × 0.3)

where the following definitions are used:

F (Frequency Gap) = The normalized difference between the observed frequency of a keyword/topic in the Scopus dataset (2014–2025) and its expected frequency based on the field’s average growth rate. This component quantifies how underrepresented a topic is relative to the overall scientific production.

D (Density Gap) = The normalized measure of thematic isolation, calculated as the inverse of the co-occurrence network density for each keyword. Topics with low connectivity to other research areas receive higher scores, indicating a lack of integration.

C (Citation Gap) = The normalized difference between the average citation impact of publications containing a specific keyword and the field’s overall average citation per document. This component identifies topics that are highly cited (high impact) but have low publication frequency (high gap).

All variables (F, D, and C) were normalized using min–max scaling to a range of 0–100:

Normalized Value = (Raw Value − Minimum Value)/(Maximum Value − Minimum Value) × 100

The final Gap_Score ranges from 0 to 100, where the following definitions are used:

- 0–33: Low gap (well-researched topic);
- 34–66: Moderate gap (partially addressed topic);
- 67–100: High gap (severely under-researched topic, requiring urgent attention).

For the period-specific analysis (e.g., 2015–2016, 2015–2025), the Gap_Score was recalculated considering the temporal distribution of publications, assigning higher weights to topics that showed declining trends over time. The Gap_Category classification (Underexplored Keywords, Funding Gap, Unexplored Combinations, Declining Topics, Methodology Gap) was determined based on the thematic cluster analysis from VOSviewer and the qualitative assessment of the research context.

The ‘Funding Gap’ category was derived from a two-step process. First, the Scopus ‘Funding Details’ metadata field (which includes sponsor name, acronym, grant number, and funding text extracted from acknowledgment sections) was systematically reviewed for all publications in the dataset. Second, keywords associated with ‘physicochemical properties’ and ‘biocompatibility’ were cross-referenced against publications containing funding information. The 0.0% funding rate reported for these topics reflects the absence of any funding acknowledgments in the publications specifically addressing these research areas, within the analyzed Scopus (2014–2025). This finding is consistent with the documented challenges of obtaining complete and reliable funding metadata in bibliometric databases, where funder information is often incomplete or inconsistently recorded.

## 3. Results and Discussion

**Figure 4:**
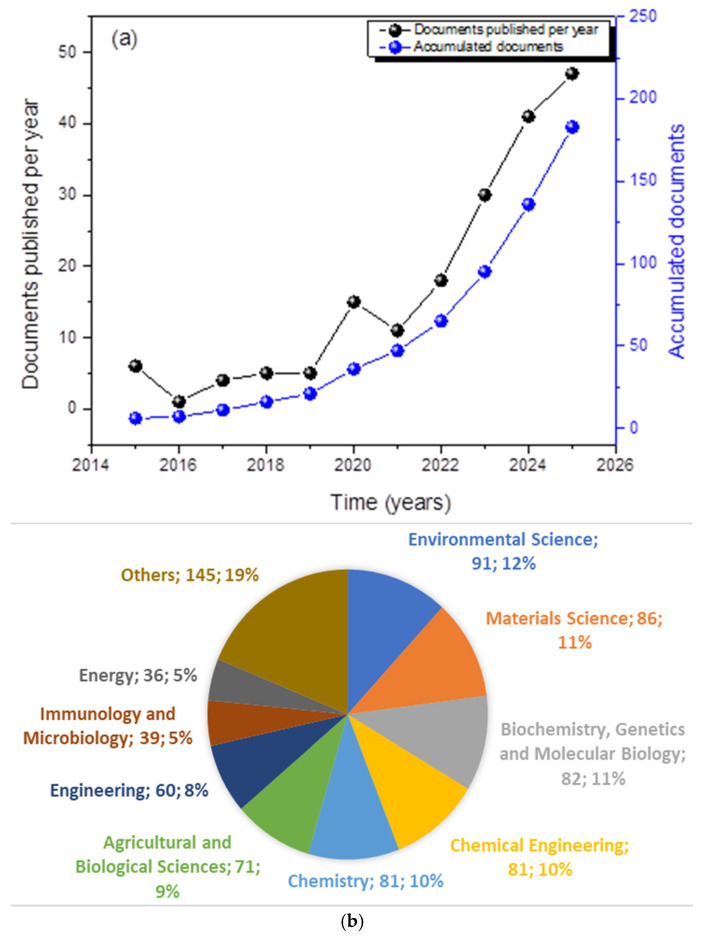
(**a**) Exponential fit of the growth of the annual and cumulative scientific production of documents on Global research and (**b**) percentage distribution of said publications by subject areas according to the Scopus classification.

The global bibliometric analysis on research trends in polyhydroxyalkanoate (PHA)-producing microorganisms reveals a picture of dynamic growth and a marked interdisciplinarity in the period 2014–2025. When evaluating scientific production (Figure 4a), it can be seen that both annual and cumulative production follow a statistically sound exponential adjustment. When contrasting these findings with the recent literature, a notable increase in publications is observed from 2021 onwards, reflecting a revitalized academic and industrial interest in sustainability after the COVID-19 pandemic [36]. This trend responds to the urgent need to develop biotechnological alternatives to conventional petroleum-based plastics [1]. Consequently, this boom shows that the optimization of microbial metabolic pathways and the use of agro-industrial residues have been consolidated as strategic priorities in the global research agenda [37].

On the other hand, the percentage distribution of the documents by thematic areas, according to the Scopus classification (Figure 4b), shows the multidisciplinary perspective of this field. Thus, it is observed that PHA research maintains a strong anchorage in the basic sciences, with a clear predominance of areas such as Immunology and Microbiology (22.5%) and Biochemistry. Genetics and Molecular Biology (19.5%). This pattern is logical, given that PHA synthesis is a fundamentally microbiological phenomenon that occurs as a response to nutritional stress, requiring a deep understanding of metabolic pathways and gene regulation [33]. However, the growing participation of Environmental Sciences (17.3%) and Chemical Engineering (10.1%) underlines the systemic approach of the sector, which ranges from the assessment of the environmental impact and biodegradability of PHAs to industrial scale-up in bioreactors and downstream processing of the material [38]. In this new stage, microbiological knowledge is articulated with the optimization of biorefineries, life cycle assessment and the development of ecological extraction methods, which confirms a maturation of the field from studies focused on individual strains to the optimization of complete industrial processes [36]. Together, the quantitative and qualitative data of the figures demonstrate that PHA synthesis is not only an object of microbiological study, but a mature and rapidly expanding technological platform.

**Figure 5:**
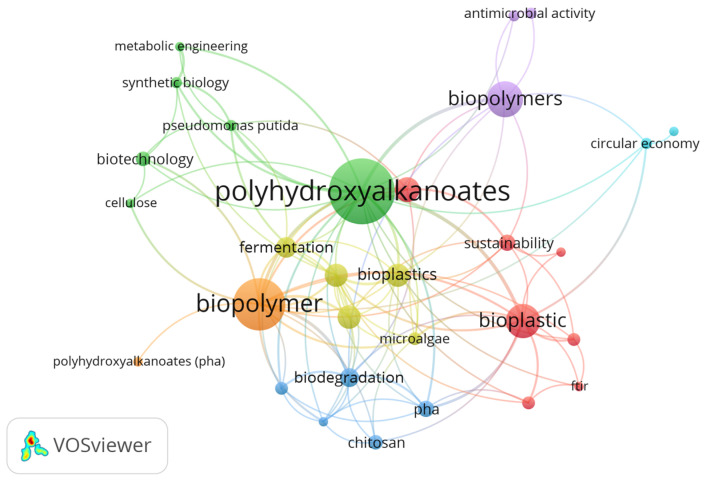
Network map of keyword co-occurrence and thematic cluster segmentation.

The patterns of co-occurrence observed in Figure 5 show that research on polyhydroxyalkanoates (PHAs) is articulated within a dense, multidisciplinary and highly interconnected thematic network. While the term “polyhydroxyalkanoates” constitutes the central node, a critical finding in the network topology is the consolidation of *Pseudomonas putida* as a model organism, displacing the traditional historical approach focused on *Cupriavidus necator.* This divergence is not a bibliometric artifact, but a reflection of real scientific trends based on the metabolic versatility of *P. putida* to synthesize medium-chain PHAS (MCL-PHA) from diverse substrates, as well as its high tolerance to oxidative stress. The recent literature confirms that the adoption of advanced synthetic biology and metabolic engineering tools particularly gene-editing systems such as CRISPR-Cas9—has made it possible to precisely redirect carbon streams on this cellular platform, facilitating the production of customized and industrially optimized polymers [39,40].

On the other hand, the strong association between the terms “biopolymer”, “bioplastic” and “biodegradation” underlines that the scientific interest is not limited to the synthesis of the material, but encompasses a critical concern for the management of its life cycle within the framework of the circular economy [41]. However, the data reveal that the real biodegradability of PHAs should not be assumed in a generic way, as it is strongly conditioned by crystallinity, the molecular composition of the polymer and the variables of the environmental environment. This challenge has driven emerging trends detected in the network, such as the development of hybrid biomaterials by interrelating with “chitosan” and “microalgae”, as well as the use of FTIR spectroscopy for precise structural characterization. Likewise, the link between “biopolymers” and “antimicrobial activity” denotes a transition towards obtaining advanced functional materials with high-value-added applications in biomedicine and smart packaging [42].

Overall, the bibliometric analysis shows that the study of PHAs has undergone a significant epistemic maturation, moving from a strictly microbiological and descriptive approach to an integrative technological platform. The current landscape merges downstream process engineering, waste valorization and environmental impact assessment, consolidating PHAs as a pillar of modern biotechnology [43]. This evolution demonstrates that the future of the sector depends on the ability to articulate precision genetic manipulation with the techno-economic and ecological viability of processes, bridging the gap between basic laboratory science and industrial scalability.

The analysis of the intellectual structure in the biotechnological production of polyhydroxyalkanoates (PHAs) reveals a clear epistemic transition from its strictly microbiological foundations to advanced applications with high added value (Table 1). Historical synthesis works, such as that of Muhammadi et al. (2015) [44], consolidated the initial conceptual framework of the field [36]; however, the recent literature evidences a critical paradigm shift. PHAs are no longer perceived merely as substitutes for conventional plastics of petrochemical origin, but as functional materials with intrinsic bioactive properties. This trend, led by research in nanomedicine (Gahlawat et al., 2016; Akolpoglu et al., 2020), demonstrates that PHA microbial nanoparticles possess antibacterial activity against critical pathogens such as Vibrio cholerae [45,46]. In this way, the economic viability of the sector no longer depends exclusively on large-scale cost competition, but on the exploitation of the biocompatibility of these polymers in biomedical markets where profit margins are significantly higher. In terms of industrial sustainability and the circular economy, reducing the cost of carbon substrate, which accounts for up to 50% of total operating expenditure, remains a strategic priority [47]. To address this limitation, current research diverges in two complementary biotechnological directions. On the one hand, the valorization of complex agro-industrial by-products (such as date syrup or whey) by means of mixed microbial consortia (MMCs) is emerging as an efficient route for mass processes, managing to reduce initial processing costs by 50% without requiring strict sterility conditions [36,48,49]. On the other hand, applications that demand high purity opt for pure cultures modified by systems biology. Precision engineering in cellular platforms such as *Escherichia coli* or *Halomonas bluephagenesis* using tools such as CRISPR-Cas9 and metabolic models at the genomic scale has made it possible to maximize the flow of carbon into the accumulation of the biopolymer in industrial bioreactors, optimizing the mechanistic efficiency of the process [50,51,52].

Despite these substantial advances in the upstream fermentation phase, downstream processing (DSP) remains the most critical bottleneck to the overall commercial viability of PHAs. Being an intracellular polymer, its traditional extraction has relied on energy-intensive methods and the use of highly toxic chlorinated organic solvents, which contradicts the principles of green chemistry [53,54]. Faced with this challenge, the scientific literature calls for an urgent transition towards ecological methodologies. Emerging innovations such as spontaneous osmotic lysis in halophilic archaea, the use of supercritical fluids, or aluminum sulfate-assisted coagulation-flocculation have proven to be able to mitigate the global warming potential of the process by 25% [55]. Therefore, the consolidation of PHAs as a mature technological platform will depend on the ability to holistically integrate eco-design in the purification stage, ensuring that the environmental balance of the entire life cycle is truly sustainable.

Table 2 shows that the analysis of the scientific leadership structure in the field of polyhydroxyalkanoates (PHAs) reveals that quantitative productivity does not linearly translate into a higher qualitative or strategic impact. This dissonance is evident when contrasting profiles with high-volume production (such as Freitas F. in Portugal) with researchers who achieve a disproportionate citation rate with fewer papers (such as Roy I.). More broadly, this phenomenon explains the “dispersed productivity paradox” observed in Asian powers such as India and China. Despite leading the total volume of publications at the country level, scientific production is fragmented among a massive network of researchers instead of concentrating on individual hegemonic figures [36]. These patterns demonstrate that true leadership in biopolymer science is determined by the ability of research groups to articulate hyper-specialised regional collaborative clusters such as the UK ecosystem focused on the valorisation of waste from the brewing industry capable of coupling knowledge generation with local industrial supply chains.

From a critical perspective, the speed of citation and impact of cutting-edge researchers lies in their direct focus on the techno-economic “pain points” of the discipline. In particular, downstream processing (DSP) continues to account for 30% to 60% of total manufacturing costs, consolidating itself as the main bottleneck in the sector. The urgency to overcome this barrier has shifted interest from purely descriptive studies of microbial screening to disruptive innovations in the intracellular recovery phase, such as coagulation-flocculation, induced osmotic lysis in halophilic archaea and the use of green solvents [36,54,55]. At the same time, the economic optimization of the upstream requires a conceptual change in the selection of substrates: the transition to agro-industrial waste rich in fermentable carbohydrates (maltose and glucose) not only reduces dependence on first-generation carbon sources, but also makes possible the mass balances necessary to scale up processes in real biorefinery environments.

Finally, emerging lines of research redefine the frontiers of environmental biotechnology through two complementary strands: adaptive bioprospecting and frontier synthetic biology. On the one hand, the selection of native microorganisms in contaminated anthropogenic niches makes it possible to exploit dual metabolic pathways capable of degrading conventional plastics and synthesizing PHAs simultaneously, integrating bioremediation with waste recovery. On the other hand, the Japanese school of thought (Higuchi-Takeuchi M. and Numata K.) is pushing the boundaries of the discipline by designing marine photosynthetic platforms that use CO2 as the primary source of carbon to co-produce advanced high-value materials, such as spider silk. The convergence of these technologies with microbial electrosynthesis positions PHAs not only as biodegradable substitutes, but as strategic vectors for carbon capture and utilization (CCU). In conclusion, the consolidation of PHAs as pillars of the global bioeconomy of the 21st century will depend on a tripartite strategy that balances the mitigation of the cost of DSP, the use of circular regional consortia and the exploitation of metabolic flexibility in the face of waste gases.

**Table 1:** Top 15 Most Cited Publications.

	Title	Authors	Cited	Year	Publisher	Type	Source Title
1	Bacterial polyhydroxyalkanoates-eco-friendly next generation plastic: Production, biocompatibility, biodegradation, physical properties and applications [44].	Muhammadic et al.	325	2015	Taylor and Francis Ltd.	Article	Green Chemistry Letters and Reviews
2	Microbial glycolipoprotein-capped silver nanoparticles as emerging antibacterial agents against cholera [45].	Gahlawat G et al.	130	2016	BioMed Central Ltd.	Article	Microbial Cell Factories
3	High-Yield Production of Biohybrid Microalgae for On-Demand Cargo Delivery [46].	Akolpoglu M.B. et al.	128	2020	John Wiley and Sons Inc.	Article	Advanced Science
4	Recycling of waste streams of the biotechnological poly(hydroxyalkanoate) production by *Haloferax mediterranei* on whey [48]	Koller M.	103	2015	Hindawi Limited	Article	International Journal of Polymer Science
5	Bioplastic (poly-3-hydroxybutyrate) production by the marine bacterium Pseudomonas xiamenensis through date syrup valorization and structural assessment of the biopolymer [49]	Mostafa Y.S.; et al.	104	2020	Nature Research	Article	Scientific Reports
6	Metabolic engineering of *Escherichia coli* for the synthesis of polyhydroxyalkanoates using acetate as a main carbon source [50]	Chen J. et al.	100	2018	BioMed Central Ltd.	Article	Microbial Cell Factories
7	Extraction of polyhydroxyalkanoates from mixed microbial cultures: Impact on polymer quality and recovery [53]	Samori C. et al.	115	2015	Elsevier Ltd.	Article	Bioresource Technology
8	A sustainable approach for the downstream processing of bacterial polyhydroxyalkanoates: State-of-the-art and latest developments [54]	Pérez-Rivero C. et al.	121	2019	Elsevier B.V.	Article	Biochemical Engineering Journal
9	Trends in mitigation of industrial waste: Global health hazards, environmental implications and waste derived economy for environmental sustainability [56]	Sharma P. et al.	125	2022	Elsevier B.V.	Article	Science of the Total Environment
10	Biosynthesis of planet friendly bioplastics using renewable carbon source [57]	Jain R. et al.	117	2015	BioMed Central Ltd.	Article	Journal of Environmental Health Science and Engineering

**Table 2:** Ranking of leading authors by publication count, citation metric, and H-index.

Rank	Author	Nº Papers	Total Citations	Avg Citations	H-Index	Country	Most Cited Publication Title
1	Freitas F.	5	60	12	4	Portugal	Exploring Microorganisms from Plastic-Polluted Sites: Unveiling Plastic Degradation and PHA Production Potential [58]
2	Roy I.	3	132	44	3	Egypt	A sustainable approach for the downstream processing of bacterial polyhydroxyalkanoates: State-of-the-art and latest developments [54]
3	Tsouko E.	4	78	19.5	3	United Kingdom	Bioconversion of underutilized brewing by-products into bacterial cellulose by a newly isolated *Komagataeibacter rhaeticus* strain: A preliminary evaluation of the bioprocess environmental impact [59]
4	Pilafidis S.	4	78	19.5	3	United Kingdom	Bioconversion of underutilized brewing by-products into bacterial cellulose by a newly isolated *Komagataeibacter rhaeticus* strain: A preliminary evaluation of the bioprocess environmental impact [59]
5	Kourmentza K.	3	77	25.67	3	United Kingdom	Bioconversion of underutilized brewing by-products into bacterial cellulose by a newly isolated *Komagataeibacter rhaeticus* strain: A preliminary evaluation of the bioprocess environmental impact [59]
6	Higuchi-Takeuchi M.	3	75	25	3	Japan	A marine photosynthetic microbial cell factory as a platform for spider silk production [60]
7	Numata K.	3	75	25	3	Japan	A marine photosynthetic microbial cell factory as a platform for spider silk production [60]
8	Sarris D.	3	71	23.67	2	United Kingdom	Bioconversion of underutilized brewing by-products into bacterial cellulose by a newly isolated *Komagataeibacter rhaeticus* strain: A preliminary evaluation of the bioprocess environmental impact [59]
9	Nikodinovic-Runic J.	3	49	16.33	3	Portugal	Upcycling biodegradable pva/starch film to a bacterial biopigment and biopolymer [61]
10	Prieto M.A.	3	46	15.33	3	Spain	A model-driven approach to upcycling recalcitrant feedstocks in *Pseudomonas putida* by decoupling PHA production from nutrient limitation [62].

Table 3 reveals the analysis of the geography of innovation around polyhydroxyalkanoate-producing microorganisms (PHAs) reveals a global picture defined by divergent but deeply complementary national strategies. The distribution of scientific production allows us to identify two clearly differentiated development models. On the one hand, India is positioned as the absolute leader in quantitative volume, concentrating 12.89% of world production. This leadership is not fortuitous, but the result of deliberate government policies such as the Anusandhan National Research Foundation (ANRF) aimed at capitalizing on large agricultural base rich in agroindustrial residues (sugarcane bagasse, rice straw) for the valorization of low-cost carbon sources [31]. On the other hand, the United Kingdom personifies a qualitative model based on transnational integration. With an international collaboration rate of 91.3% and an exceptional average of 29.43 citations per article, British excellence demonstrates that multi-lingual collaboration and strict evaluation frameworks (such as the Research Excellence Framework) act as critical drivers for scientific impact, compensating for a lower volume of gross output through access to state-of-the-art infrastructures in synthetic biology and process modelling [63,64].

By contrasting these patterns with the recent literature, nuances emerge that enrich the discussion on the evolution of the field. While previous studies agree on the quantitative dominance of Asian powers such as India and China in specific niches such as halophilic bacteria [5], the analysis of the period 2014–2025 provides greater granularity by identifying the emergence of new European centres of excellence. In this context, Italy’s consolidation in second place in the world (28 documents) and with a solid rate of international collaboration (CCM of 53.57%) marks a departure from the historical trends that placed the United States and Germany exclusively at the forefront [47]. This Italian rise reflects a strategic shift supported by the European Union’s circular economy policies, shifting the traditional approach focused on model organisms such as *Cupriavidus necator* towards harnessing indigenous microbial diversity and integrated treatment of activated sludge and municipal organic waste [36].

From a biotechnological and industrial perspective, the globally distributed nature of PHA research implies that the commercial viability of the biopolymer will not depend on a single nation, but on an effective transfer of interregional knowledge. India has the structural advantages to lead the production of large-scale, low-cost PHAs due to the massive availability of residual raw materials, a critical factor in competing with petrochemical-based plastics [31]. However, the translation of this biomass into advanced materials requires the precision metabolic engineering tools developed in the United Kingdom [63] and the process integration capability demonstrated by Italy. Likewise, the case of Portugal illustrates how countries with lower gross volumes can achieve a disproportionate impact (H-index of 11) through the hyper-specialization of centers such as iBET in critical niches such as environmental bioprospecting and the optimization of downstream processing [58]. In conclusion, synergy between these diverse national ecosystems is the prerequisite for PHAs to overcome their current economic bottlenecks and consolidate themselves as a mature and competitive pillar of the global bioeconomy.

Table 4 shows the analysis of metabolic specialization in polyhydroxyalkanoate (PHA)-producing microorganisms reveals a direct and fundamental correlation between cell phylogeny, the active biosynthetic pathway and the thermomechanical properties of the resulting polymer. This taxonomic heterogeneity, which ranges from Gram-negative bacteria (*Cupriavidus necator*, *Pseudomonas putida*) and Gram-positive bacteria (*Bacillus megaterium*) to halophilic archaea (*Haloferax mediterranei*), determines the window of applicability of the biomaterial in critical sectors such as biopackaging. While the canonical pathway (PhaA-PhaB-PhaC) in producers of short-chain PHA (scl-PHA), such as *Alcaligenes latus* and *C. necator*, generates rigid and highly crystalline homopolymers such as polyhydroxybutyrate (PHB) that are ideal for rigid packaging, alternative pathways coupled to beta-oxidation and de novo synthesis of fatty acids in Pseudomonas channel intermediates towards medium-chain PHA polymerization (mcl-PHA) with superior flexibility [65,66]. Likewise, exceptional cases such as that of *Paracoccus denitrificans*, capable of exclusively synthesizing the homopolymer P(3HV), demonstrate that the bioprospecting of specific ecological niches allows access to macromorphological architectures with improved processability and ductility, overcoming the intrinsic limitations of conventional PHB [67].

By contrasting these patterns with the recent literature, nuances emerge that enrich the discussion on the evolution of the field. While previous studies agree on the quantitative dominance of Asian powerhouses such as India and China in specific niches such as halophilic bacteria [5], the analysis of the period 2014–2025 provides greater granularity by identifying the emergence of new European centres of excellence. In this context, Italy’s consolidation in second place in the world (28 documents) and with a solid international collaboration index (CCM of 53.57%) marks a departure from historical trends that placed exclusively the United States and Germany at the forefront [47]. This Italian rise reflects a strategic shift supported by the European Union’s circular economy policies, shifting the traditional approach focused on model organisms such as *Cupriavidus necator* towards harnessing indigenous microbial diversity and integrated treatment of activated sludge and urban organic waste [36].

From a biotechnological and industrial perspective, the globally distributed nature of PHA research implies that the commercial viability of the biopolymer will not depend on a single nation, but on an effective transfer of interregional knowledge. India possesses the structural advantages to lead in large-scale, low-cost PHA production due to the massive availability of waste raw materials, a critical factor in competing with petrochemical-based plastics [31]. However, the translation of this biomass into advanced materials requires the precision metabolic engineering tools developed in the United Kingdom [64] and the process integration capability demonstrated by Italy. Likewise, the case of Portugal illustrates how countries with lower gross volume can achieve a disproportionate impact (H-index of 11) through the hyper-specialization of centers such as iBET in critical niches such as environmental bioprospecting and the optimization of downstream processing [58]. In conclusion, synergy between these diverse national ecosystems is the prerequisite for PHAs to overcome their current economic bottlenecks and consolidate themselves as a mature and competitive pillar of the global bioeconomy.

Although Table 4 focuses primarily on bacterial producers of PHA due to their historical predominance in the literature, emerging research highlights the important potential of halophilic archaea as sustainable cellular factories. Among them, *Haloferax mediterranei* stands out as a promising candidate for industrial-scale PHA production, achieving PHBV yields of up to 77.8 g/L from extruded corn starch and 5.5 g/L from hydrolyzed whey under non-sterile conditions, accumulating up to 58.4% PHB of its cell dry weight [68] The unique advantages of halophilic archaea, such as lower risk of contamination, simplified downstream processing via osmotic lysis, and tolerance to inhibitory compounds present in residual substrates, position them as key players in the future of sustainable bioplastics biotechnology.

**Table 4:** PHA-Producing Microorganisms and Their Potential Applications in Biopackaging.

Genus/Species	PHA Type Produced	Reported Substrate	Maximum PHA Content (% Cell Dry Weight)	Potential Biopackaging Application	Reference
*Cupriavidus necator* (formerly *Ralstonia eutropha*)	PHB, PHBV, P(3HB-co-4HB)	Glucose, sucrose, glycerol, vegetable oils, organic acids	>80%	Rigid packaging, bottle caps, thermoformed containers, technical films, and textile fibers	Sudesh et al. (2011); Koller et al. (2017) [12,69]
*Ralstonia eutropha*	PHB, PHBV	Soybean oil, organic waste-derived fatty acids, glucose	~80%	Polymeric blends and reinforced biodegradable packaging materials	Park & Kim (2011) [70]
*Halomonas bluephagenesis* TD0	PHB, P(3HB-co-4HB), mcl-PHA	Glucose, seawater-based medium, sucrose, glycerol	80%	Flexible films for food packaging and coating applications	Tan et al. (2011, 2014) [71]
*Azotobacter vinelandii*	PHB	Crude glycerol, glucose, fructose	70–80%	Biodegradable films and agricultural support materials	Yoshida et al. (2022) [72]
*Bacillus megaterium*	PHB, PHBV	Date syrup, food-processing residues, glucose, sucrose	50–85%	Biodegradable coatings, rigid packaging, and medical-grade containers	Omar et al. (2001); Shrivastav et al. (2013) [73,74]
*Pseudomonas putida*	mcl-PHA	Heptanoate, fatty acids, vegetable oils, styrene	71%	Flexible films, adhesives, elastomers, and specialty coatings	Wang et al. (2009); Prieto et al. (2016) [13,75]
*Pseudomonas oleovorans*	mcl-PHA	Alkanes, octanoate, vegetable oils	30–50%	Water-resistant flexible films and functional coatings	Preusting et al. (1993) [76]
*Paracoccus denitrificans*	PHB	Methanol, acetate, fatty acids, corn stover hydrolysate	~50%	Industrial rigid packaging and short-life disposable containers	Sawant et al. (2015) [77]
*Burkholderia sacchari*	PHB, PHBV	Wheat straw hydrolysate, sucrose, lignocellulosic residues	60–70%	Biopackaging for dry products, protective films, and biodegradable coatings	Cesário et al. (2014) [78]
*Alcaligenes latus*	PHB	Sugar beet juice, glucose, sucrose	88%	Single-use food containers and rigid biodegradable packaging	Yamane et al. (1996) [79]
*Haloferax mediterranei*	PHBV	Hydrolyzed whey, food waste digestate, extruded cornstarch	58.4%	Flexible films, food packaging, biomedical devices; non-sterile cultivation reduces costs	Zhang et al.(2025) [68]

## 4. Future Research Trends

The systematic analysis of knowledge gaps in the production of polyhydroxyalkanoates (PHAs) reveals that downstream processing (DSP) is consolidated as the most severe economic barrier to the commercial viability of the biopolymer (Table 5). Currently, DSP accounts for 30% to 60% of the total cost of production, which explains why PHAs exhibit a restrictive market price of $4.96 to $6.06 per kg, unable to compete with the $1.32 to $1.92 per kg cost of conventional petrochemical plastics [80]. This limitation is a direct consequence of the intracellular nature of the polymer, the recovery of which requires mechanical, chemical, or enzymatic methods of high energy intensity or dependent on toxic chlorinated solvents such as chloroform [81,82]. Overcoming this bottleneck by eliminating high energy consumption projects an estimated 18% to 30% reduction impact on total costs [81]. To this end, it is mandatory to redirect R + D efforts towards sustainable extraction technologies, such as coagulation-flocculation [55], the use of green solvents such as gamma-valerolactone [5,83] or osmotic lysis in extreme systems, while simultaneously guaranteeing the structural purity required in high-value niches such as biomedicine, functional biosensors and hospital antimicrobial coatings [5,84].

In the field of bioprocess engineering, the transition to a viable circular economy depends on the scalability of mixed microbial consortia (MMCs), which offer the theoretical advantage of valorizing non-sterile waste substrates such as palm oil or fish oil effluents [14,85]. However, current research remains mostly confined to pure lab-scale cultures, facing inherent instability in large-scale bioreactors [80,83]. This instability has clear mechanistic foundations: competition for nutrients, the accumulation of inhibitory metabolic intermediates, and genetic drift, where natural selection disadvantages accumulating strains over fast-growing strains (Ahuja et al., 2024) [86]. While strategies such as feast-famine cycles mitigate this phenomenon, their industrial dynamic control (rate of organic charge and dissolved oxygen) remains a major technical challenge [86]. This problem is aggravated by the ambiguity in the literature on the term “pilot scale”, whose volumes fluctuate drastically between 30 L and 5000 L, preventing a rigorous quantitative evaluation [87]. Thus, despite advances with recombinant strains such as *Halomonas bluephagenesis* capable of accumulating up to 85% PHA, productivities under non-sterile conditions (1.0–3.0 g/L/h) continue to be below full commercial viability levels [80], detracting from the consistency of massive activated sludge platforms that theoretically yield up to 65% by dry weight [86].

To transcend the stagnation of classical analytical approaches, the scientific agenda must pivot towards precision synthetic biology and the comprehensive exploration of the so-called “PHAome”. Given that only 15% of extremophile archaeal diversity has been exploited, bioprospecting of understudied taxa such as polar bacteria or Actinobacteria represents a critical frontier to synthesize monomers with superior elastomeric properties and enable fermentation processes free of costly sterilization, whose overall profitability can be substantially improved by the co-production of high-value carotenoids [2,5]. Simultaneously, gene editing tools such as CRISPR-Cas9 and the use of carbon nanodots emerge as disruptive alternatives to optimize carbon flux in microalgae and bacteria [33,88]. An emerging line of research, although still exploratory, is the application of quantum-type decision-making models to simulate the complex regulatory dynamics of PHA biosynthesis. Ho et al. (2024) proposed a quantum-type model that encodes gene expression and regulatory events as hidden layers by the general transformation of a density matrix, demonstrating its application to the production of mcl-PHA in *Pseudomonas putida* under different C/N ratios [89]. This approach, inspired by previously developed quantum-like models for gene regulation in *E. coli* (Asano et al., 2012), offers a novel perspective for capturing the uncertainty inherent in metabolic regulatory networks [90]. However, it is important to note that this remains a nascent area of research, with limited validation and replication studies currently available. The potential integration of quantum modeling with predictive computational frameworks, including genomic-scale metabolic models (GEMs) and machine learning algorithms, warrants further exploration, but should be considered a long-term frontier rather than an immediate trend.

In short, the consolidation of PHAs as pillars of the global bioeconomy requires the unification of multi-omics data (genomics, transcriptomics and proteomics) with these advanced models, transforming isolated observations into robust predictive tools capable of simulating and controlling complex fermentation scenarios on an industrial scale [5].

Table 6 reveals a troubling misalignment in research on polyhydroxyalkanoate (PHA)-producing microorganisms, where the Gap_Category of “Underexplored Keywords” identifies “physicochemical properties” and “biocompatibility” with the highest Gap_Score (228.7) of the 2015–2025 Research_Period. This gap is directly linked to a “Funding Gap” of 0.0% funding for these very areas, which is alarming given that technical characterization is the cornerstone for the transition of bioplastics into the industrial and biomedical markets. Likewise, the “Unexplored Combinations” category presents a score of 195.8, suggesting that the interdisciplinary integration between “PHA biosynthesis” and its “applications” remains fragmented, prioritizing laboratory production over material performance in real-world scenarios.

There is a concerning absence of funding acknowledgments (0.0%) for characterizing physicochemical properties and biocompatibility within the analyzed Scopus corpus (2014–2025). This finding was obtained by cross-referencing publications containing these keywords against the ‘Funding Details’ metadata field in Scopus, which captures funding information from acknowledgment sections and dedicated funding statements. The complete absence of funding records for these critical research areas essential for industrial and biomedical commercialization represents a significant knowledge gap that warrants urgent attention from funding agencies. However, it is important to acknowledge the well-documented limitations of funding metadata in bibliometric databases, where funding information may be incompletely recorded or omitted by authors.

It is important to clarify that the classification of ‘PHA biosynthesis’ as an ‘Underexplored Keyword’ (Gap_Score: 115) and ‘Biosynthesis Modelling’ as a knowledge gap does not contradict the well-established biochemical understanding of the PHA biosynthetic pathway. The canonical three-enzyme pathway (PhaA, PhaB, PhaC) has been extensively characterized over decades. Rather, these gaps reflect the limited application of systems-level approaches such as genome-scale metabolic models (GEMs), integrative multi-omics analysis, and predictive computational modeling to optimize carbon flux and metabolic network performance under industrially relevant conditions. While CRISPR-Cas9 and synthetic biology tools have revolutionized strain engineering, their full potential for dynamic metabolic control and real-time process optimization remains underexplored. Thus, the gap identified in this study is not in the fundamental understanding of PHA biosynthesis, but in the translational and integrative research required to achieve industrial scalability.

Another relevant finding is the identification of unexplored combinations (Gap_Score: 195.8) and the classification of agricultural residues as a declining issue (Gap_Score: 117), a trend that contrasts with the current consensus that residual substrates constitute the main strategy to reduce the cost of production of PHAs [80]. This apparent contradiction can be attributed to the growing emphasis on genetic engineering of model strains in the face of the technological complexity associated with the use of lignocellulosic residues. In parallel, limited exploration of halophilic archaea and other understudied taxa restricts access to biotechnological platforms with competitive advantages for downstream processing, such as osmotic lysis and culture without strict sterility conditions [80,91,92]. Taken together, these results indicate that the industrial consolidation of PHAs will depend less on increasing biosynthetic capacity and more on integrating microbiology, materials science, process engineering, and waste valorization within interdisciplinary strategies that allow the development of biopolymers with functional properties, competitive costs, and commercially viable applications [37,93,94].

## 5. Conclusions

The present bibliometric and scientometric study offers a comprehensive view of the global panorama of research on polyhydroxyalkanoate (PHA)-producing microorganisms between 2014 and 2025, tracing structural changes, technological frontiers, and critical knowledge gaps. The exponential increase in scientific publications and international collaboration networks underscores a deep global commitment to transition from traditional petrochemical plastics to sustainable biobased packaging alternatives within a circular economy framework. This identified thematic evolution, which moves from the basic microbial physiological characterization to highly sophisticated fields such as metabolic engineering, synthetic biology and gene editing using CRISPR-Cas9, demonstrates that the discipline is moving firmly towards technological maturity. However, to fully unlock the commercial potential of PHAs, the scientific agenda must resolve urgent systemic bottlenecks. The Gap Scores revealed that downstream processing (extraction and purification), dynamic control of mixed microbial cultures using complex industrial effluents, and the marked lack of funding for deep physicochemical and biocompatibility characterization are the main barriers in the sector. Therefore, future research directions should prioritize the transition to green extraction methods that reduce chemical and energy inputs, supported by transcontinental and multidisciplinary collaborations between academia and the biotech industry. Finally, although this model has methodological scopes limited to the selection of a single database and open access publications, the robust convergence of the indicators supports the validity of the trends identified; this projects the need for future research to integrate multiple repositories and incorporate wider time windows to consolidate the global historical panorama of PHAs.

## Figures and Tables

**Table 3:** Top Countries for the Scientific Production of Polyhydroxyalkanoate (PHA)-Producing Microorganisms: Publications, Citation Impact, and International Collaboration.

Rank	Country	Nº Publications	Frequency (%)	SCP	MCP	MCP Ratio (%)	H-Index	Total Citations	Average Article Citations
1	India	41	12.89	29	12	29.27	13	888	21.66
2	Italy	28	8.81	13	15	53.57	13	678	24.21
3	China	24	7.55	10	14	58.33	11	631	26.29
4	USA United States	23	7.23	12	11	47.83	10	379	16.48
5	UK	23	7.23	2	21	91.3	13	677	29.43
6	Brazil	21	6.6	15	6	28.57	11	206	9.81
7	Portugal	19	5.97	7	12	63.16	11	480	25.26
8	Spain	18	5.66	11	7	38.89	13	398	22.11
9	Germany	16	5.03	8	8	50	11	557	34.81
10	Egypt	15	4.72	10	5	33.33	8	285	19

**Table 5:** Summary: Knowledge Gaps and Research Frontiers.

Knowledge Gap	Description	Current Status	Importance	Key Applications	Main Barriers	Estimated Impact
Downstream Processing (DSP)	Lack of standardized, low-cost extraction methods.	DSP represents a massive portion of total costs; use of toxic solvents.	Crucial for economic competitiveness against fossil-based plastics.	Medical-grade bioplastics and industrial packaging.	High energy consumption and low purity of native granules.	18–30% reduction in production costs.
Scalability of Consortia	Instability of mixed microbial communities in large-scale reactors.	Most studies are at laboratory scale with pure cultures.	Enables the use of non-sterile waste substrates, reducing costs.	Bioremediation and industrial waste management.	Nutrient competition and accumulation of toxic intermediates.	Viability of the biotechnological circular economy.
Unexplored Taxa	Lack of knowledge about PHA potential in Archaea and polar extremophilic bacteria.	Only 15% of extremophilic archaea diversity has been exploited.	Discovery of unique monomers with novel physical properties.	High-flexibility materials and advanced biomedical applications.	Culturing difficulties and lack of specific genetic tools.	Diversification of the biopolymer portfolio (“PHAome”).
Biosynthesis Modelling	Uncertainty in the dynamic regulation of carbon metabolic flux.	Classical models limited by biological complexity.	Precise optimization of C/N ratio for maximum yield.	Automated control of smart bioreactors.	Need for advanced algorithms (AI/Quantum Computing).	20–30% increase in intracellular accumulation.

Note: ‘Biosynthesis Modelling’ refers to the current lack of integrative predictive frameworks, including genome-scale metabolic models (GEMs) and multi-omics approaches, capable of optimizing carbon flux distribution and PHA biosynthesis under industrial-scale conditions. It does not denote insufficient understanding of the fundamental biochemical pathways involved in PHA biosynthesis.

**Table 6:** Major Research Gaps, Underexplored Topics, and Future Research Directions in Global Research on Polyhydroxyalkanoate-Producing Microorganisms.

Gap_Category	Gap_Item	Research_Period	Recommendation	Gap_Score
Underexplored Keywords	physiochemical properties	2015–2025	Increase research focus on this topic	228.7
Underexplored Keywords	biocompatibility	2015–2025	Increase research focus on this topic	228.7
Funding Gap	biocompatibility	Various	Increase funding (0.0% currently funded)	212.5
Funding Gap	physiochemical properties	Various	Increase funding (0.0% currently funded)	212.5
Unexplored Combinations	applications + physio…	2015–2025	Explore interdisciplinary combinations	195.8
Unexplored Combinations	biocompatibility + biode…	2015–2025	Explore interdisciplinary combinations	195.8
Unexplored Combinations	applications + biodegrad…	2015–2025	Explore interdisciplinary combinations	195.8
Unexplored Combinations	biocompatibility + physi…	2015–2025	Explore interdisciplinary combinations	195.8
Unexplored Combinations	applications + biocompa…	2015–2025	Explore interdisciplinary combinations	195.8
Unexplored Combinations	biodegradation + physio…	2015–2025	Explore interdisciplinary combinations	195.8
Unexplored Combinations	PHA biosynthesis + appli…	2015–2025	Explore interdisciplinary combinations	195.8
Unexplored Combinations	PHA biosynthesis + bioc…	2015–2025	Explore interdisciplinary combinations	195.8
Unexplored Combinations	PHA biosynthesis + physi…	2015–2025	Explore interdisciplinary combinations	195.8
Unexplored Combinations	PHA biosynthesis + biod…	2015–2025	Explore interdisciplinary combinations	195.8
Declining Topics	Antibacterial agent	2016–2016	Revitalize or transition research direction	130
Declining Topics	Agricultural waste	2015–2016	Revitalize or transition research direction	117
Underexplored Keywords	PHA biosynthesis	2015–2025	Increase research focus on this topic	115
Underexplored Keywords	applications	2015–2025	Increase research focus on this topic	114.65
Methodology Gap	Not specified	Various	Develop and apply underutilized methodologies	112.8
Funding Gap	Recovery	Various	Increase funding (0.0% currently funded)	110.5
Funding Gap	Digestion	Various	Increase funding (0.0% currently funded)	110.5

Note: ‘PHA biosynthesis’, classified as an Underexplored Keyword, denotes a research gap in the integration of systems biology, multi-omics, and genome-scale metabolic models (GEMs) for predicting and optimizing carbon flux toward industrial-scale PHA production. It should not be interpreted as a lack of understanding of the canonical PHA biosynthetic pathway, which is well established in the literature.

## Data Availability

The original contributions presented in this study are included in the article. Further inquiries can be directed to the corresponding author.

## Abbreviations

The following abbreviations are used in this manuscript:

PHA	Polyhydroxyalkanoate
